# Supplementary material for: Reversible synaptic deficits in early-stage batten disease
Source: J Transl Med. 2026 May 20;24:695. doi: 10.1186/s12967-026-08304-w (PMC13196110; doi:10.1186/s12967-026-08304-w)
Supplement: Supplementary file 1 — Supplementary Material 1 [file 12967_2026_8304_MOESM1_ESM.pdf]

Supplementary Information

Reversible synaptic deficits in early-stage Batten disease

Masood Ahmad Wani[1,2], Chloe M. Hall[3], Thomas Mittmann[2,3], Benedikt Grünewald[1,2,†,*]
Jakob von Engelhardt[1,2,†,*],

Author affiliations:

[1]Institute of Pathophysiology, University Medical Center of the Johannes Gutenberg University Mainz, 55128 Mainz, Germany

[2]Focus Program Translational Neuroscience (FTN), University Medical Center of the Johannes Gutenberg University Mainz, 55131 Mainz, Germany

[3]Institute for Physiology, University Medical Center of the Johannes Gutenberg University Mainz, 55128 Mainz, Germany

[†] These authors contributed equally

[*]Corresponding authors:

Benedikt Grünewald, PhD
Institute of Pathophysiology, University Medical Center of the Johannes Gutenberg University Mainz Duesbergweg 6
55128 Mainz Germany
e-mail: b.gruenewald@uni-mainz.de
Phone: +49 6131 3922395

And

Prof. Dr. Jakob von Engelhardt, MD
Institute of Pathophysiology, University Medical Center of the Johannes Gutenberg University Mainz Duesbergweg 6
55128 Mainz
E-Mail: engelhardt@uni-mainz.de
Phone.: 06131 39 25005
Fax: 06131 39 25560

Contents

Supplementary Figure S1 1
Supplementary Figure S2 2
Supplementary Figure S3 3
Supplementary Figure S4 4
Supplementary Figure S5 5
Supplementary Figure S6 6
Supplementary Figure S7 7
Supplementary Figure S8 8
Table 1. Intrinsic membrane properties of DG granule cells from WT and *Cln3*$^{\Delta ex7/8}$ mice at 3 weeks and 4-months of age 9
Table 2. Synaptic transmission: mEPSC and mIPSC values of DG granule cells from WT and *Cln3*$^{\Delta ex7/8}$ mice at 3 weeks and 4 months of age 11
Table 3. AMPA/NMDA ratio values of DG granule cells from 4-month-old WT and *Cln3*$^{\Delta ex7/8}$ mice 12
Table 4. Dendritic spine density of DG granule cells from 4-month-old WT and *Cln3*$^{\Delta ex7/8}$ mice 12
Table 5. Readily-releasable pool (RRP) values of DG granule cells from 4-month-old WT and *Cln3*$^{\Delta ex7/8}$ mice13
Table 6. Hippocampal LTP in 4-month-old WT and *Cln3*$^{\Delta ex7/8}$ mice 13
Table 7. Presynaptic properties recorded from DG-GCs after AAV9-mediated CLN3 re-expression in presynaptic perforant pathway projections from Entorhinal cortex to DG in 4-month-old *Cln3*$^{\Delta ex7/8}$ mice 14
Table 8. Pre- and postsynaptic properties recorded from DG-GCs after AAV9-mediated CLN3 re-expression in postsynaptic granule cells of DG in 4-month-old *Cln3*$^{\Delta ex7/8}$ mice 15

Supplementary Figure S1

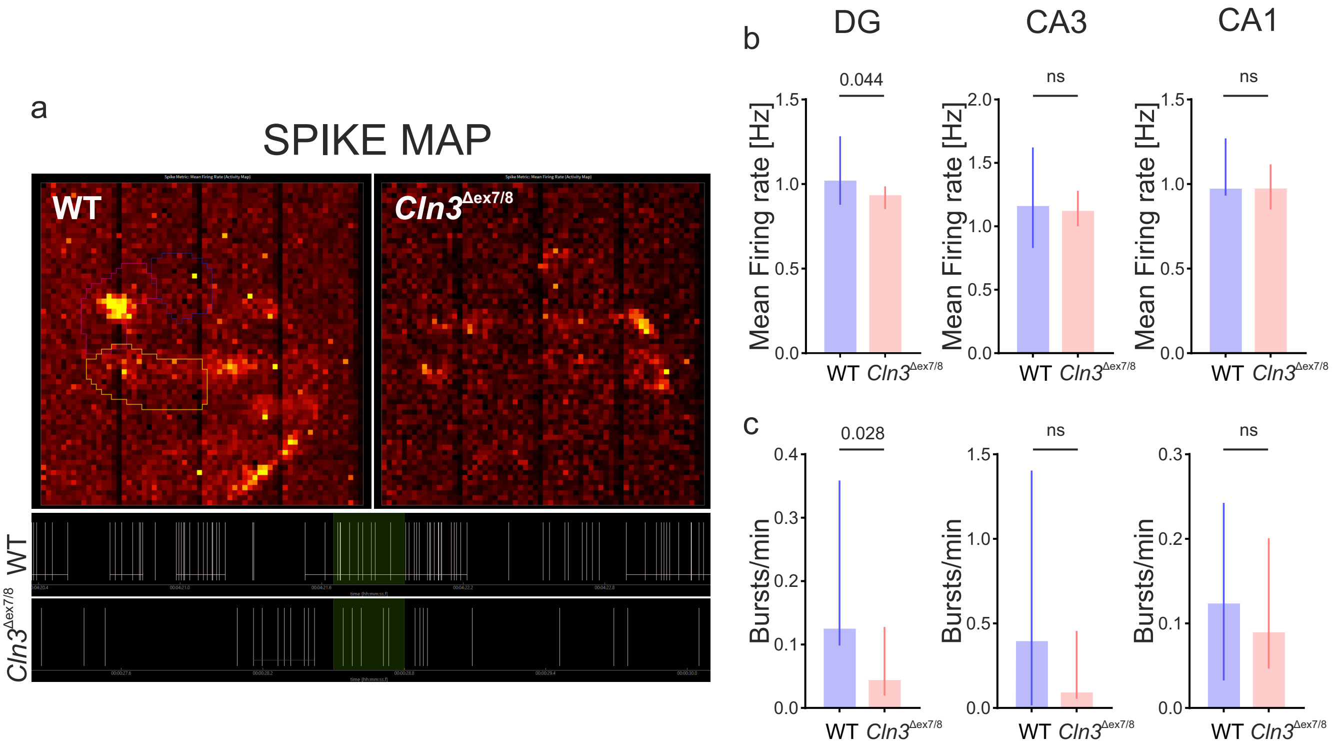

Fig. S1 Reduced network bursting in the hippocampus of *Cln3*$^{\Delta ex7/8}$ mice. (a) Representative heat map of spontaneous activity (spikes) from acute hippocampal slices (4-month-old) recorded with HD-MEA. Bursts were defined as ≥5 consecutive spikes with inter-spike interval ≤100 ms (bottom panel: detected spikes grouped into bursts). (b) Summary bar plots of mean firing rate (MFR, Hz) and (c) bursting rate (bursts/min) in DG, CA3 and CA1 for WT and *Cln3*$^{\Delta ex7/8}$ slices (n= 10, 14 slices; N= 4, 4 mice [WT, *Cln3*$^{\Delta ex7/8}$]). Data are mean [95% CI] for DG, CA3 MFR, and median [IQR] for all other bar plots; ns, not significant; p-values from Unpaired t-tests and Mann-Whitney tests

Supplementary Figure S2

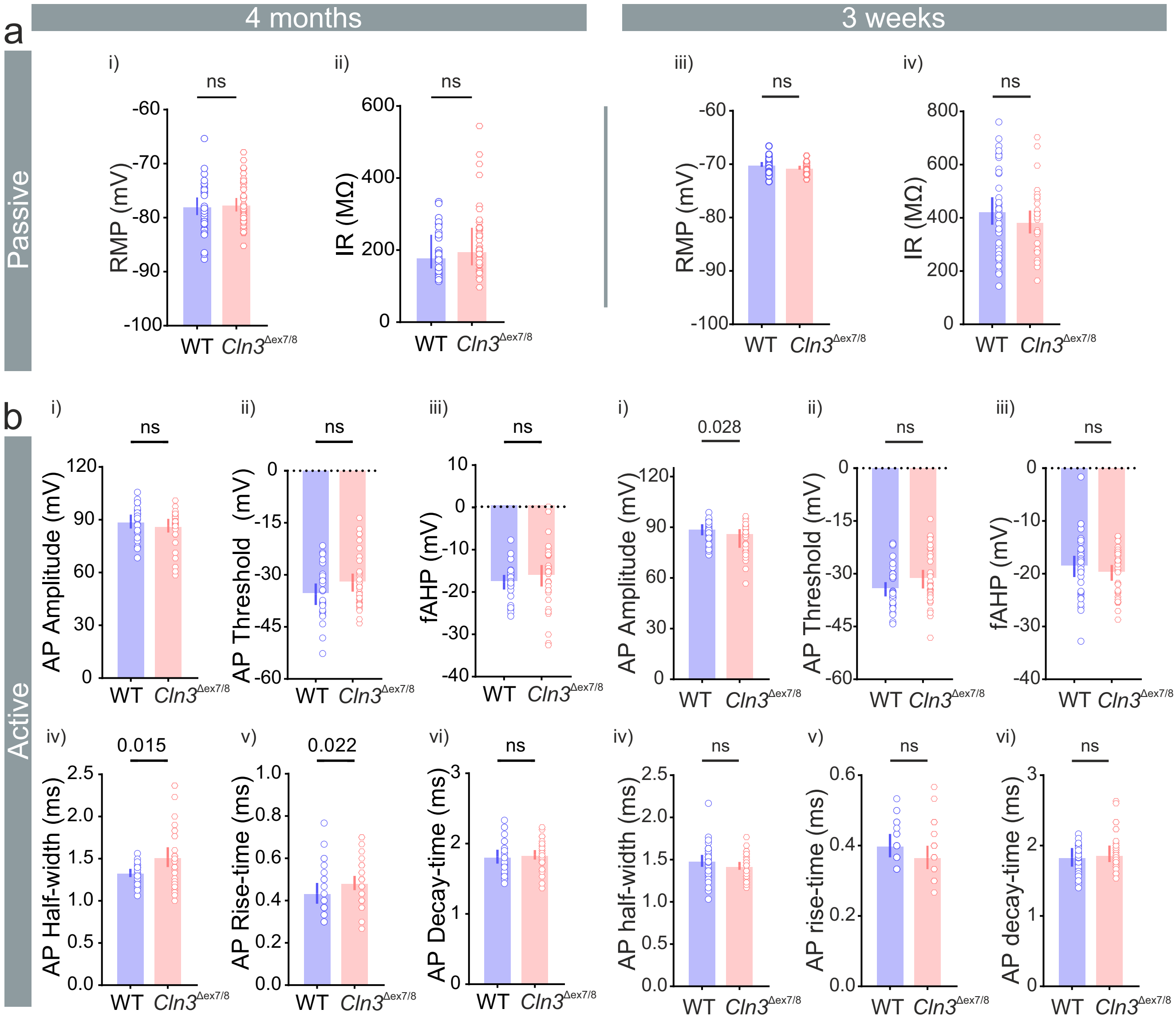

Fig. S2 Intrinsic membrane properties of neurons in *Cln3*$^{\Delta ex7/8}$ mice. (a) Passive properties: summary bar plots of resting membrane potential (RMP) and input resistance (IR) at 4-months (left, mean [95% CI] for RMP plot and median [IQR] for IR plot; n= 36, 43 cells; N= 3, 3 mice [WT, *Cln3*$^{\Delta ex7/8}$]] and at 3-weeks (right, mean [95% CI]; n= 38, 33 cells; N= 5, 4 mice [WT, *Cln3*$^{\Delta ex7/8}$]]. Statistical analysis with linear mixed-effects model. (b) Active properties: summary bar plots of action potential (AP) amplitude, threshold, fast afterhyperpolarization (fAHP), half-width, rise-time, and decay-time at 4-months (left; n= 26, 30 cells; N= 4, 5 mice [WT, *Cln3*$^{\Delta ex7/8}$]) and at 3-weeks (right; n= 27, 24; N= 5, 4 mice [WT, *Cln3*$^{\Delta ex7/8}$]). Data are mean [95% CI] for plots in 4-month (b: vi), 3-wks (b: ii, iii, iv), and median [IQR] for all other plots in (b); ns, not significant; p-values from Unpaired t-tests and Mann-Whitney tests

Supplementary Figure S3

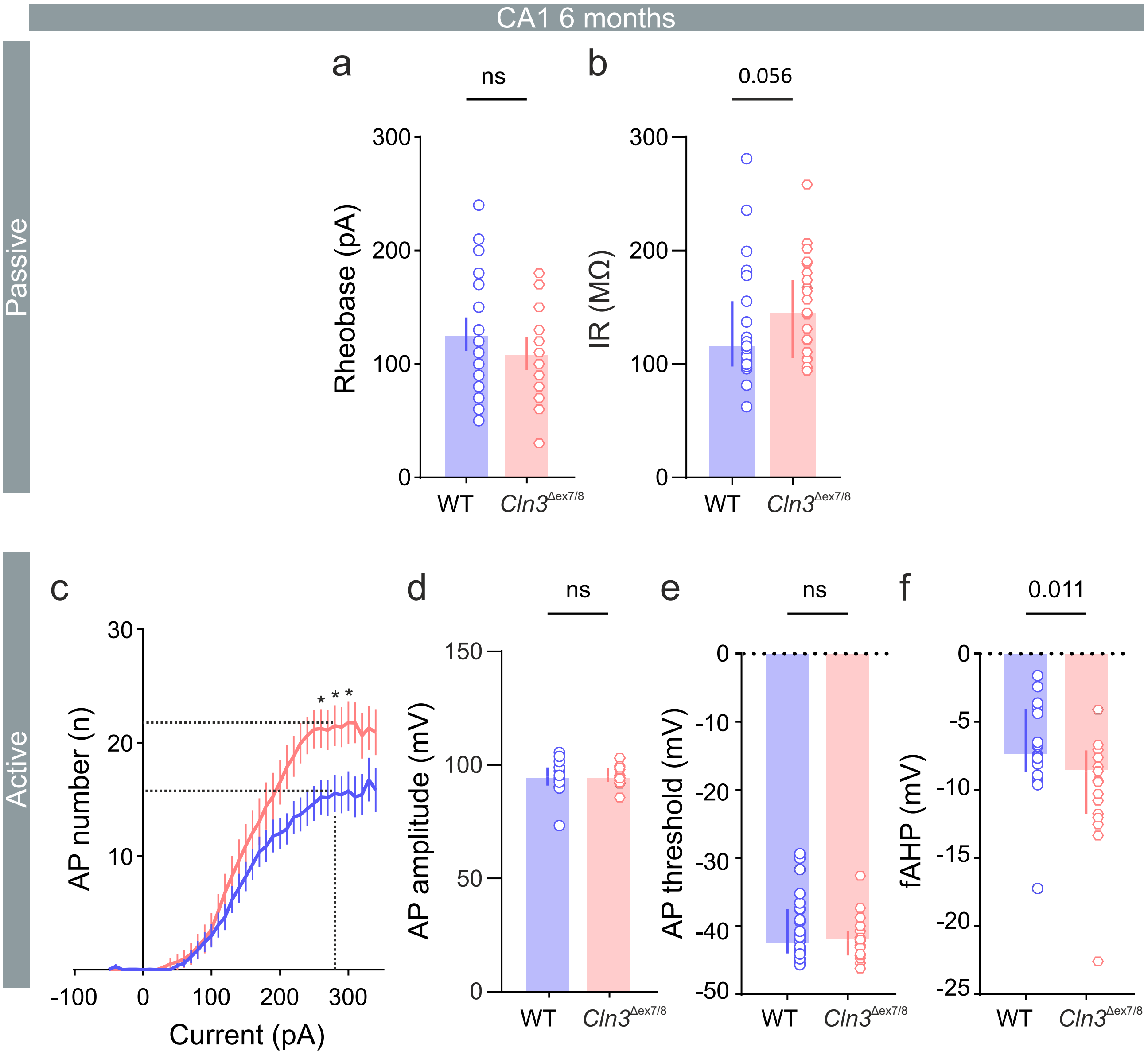

Fig. S3 Intrinsic membrane properties of CA1 neurons at 6-months in *Cln3*$^{\Delta ex7/8}$ mice. (a, b) Summary bar plots of rheobase (pA) and input resistance (IR). (c) Number of action potentials (AP) plotted against increasing current steps, mean ± SEM. (d-f) Summary bar plots of AP amplitude, threshold, and fast afterhyperpolarization [n= 31, 27 cells; N= 5, 5 mice [WT, *Cln3*$^{\Delta ex7/8}$]]. Data are mean ± SEM in (c) (*p < 0.05; Mann-Whitney tests), mean [95% CI] in (a) and median [IQR] (b, d-f); ns, not significant; p-values from Unpaired t-test (a) and Mann-Whitney test (f)

Supplementary Figure S4

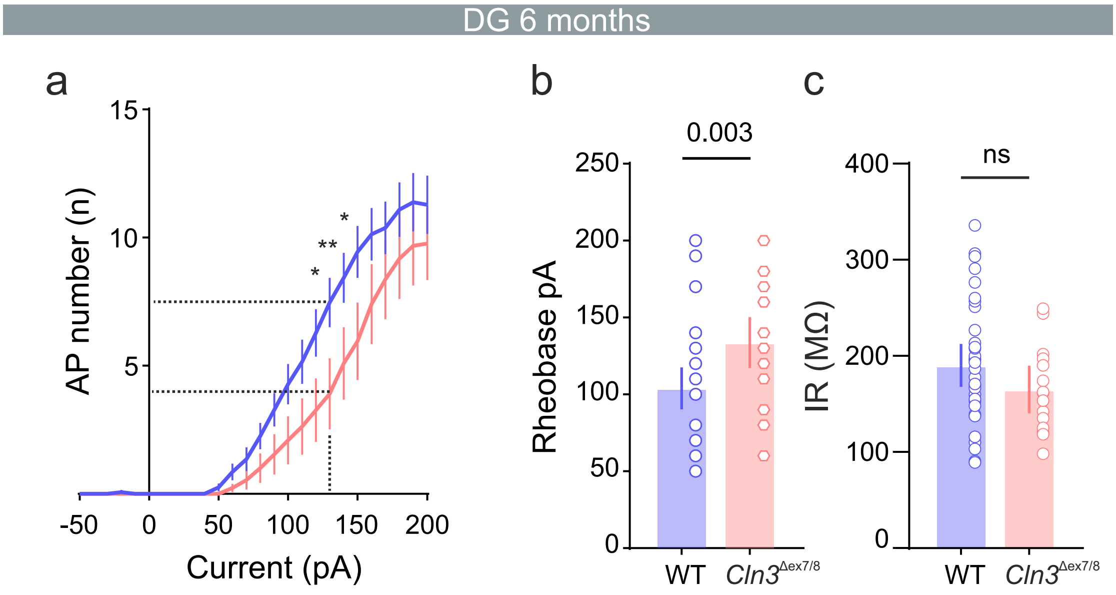

Fig. S4 Intrinsic membrane properties of DG granule cells at 6-months in *Cln3*$^{\Delta ex7/8}$ mice. (a) Number of action potentials (AP) plotted against increasing current steps, mean ± SEM. (b) Summary bar plots of Rheobase (pA) and (c) input resistance (IR). Data are mean ± SEM in (a) (**$p < 0.01$, *$p < 0.05$; Mann-Whitney tests), median [IQR] in (b) and mean [95% CI] in (c); n= 33, 21 cells; N= 5, 3 mice [WT, *Cln3*$^{\Delta ex7/8}$]); ns, not significant; p-values from Unpaired t-test (IR) and Mann-Whitney test (rheobase)

Supplementary Figure S5

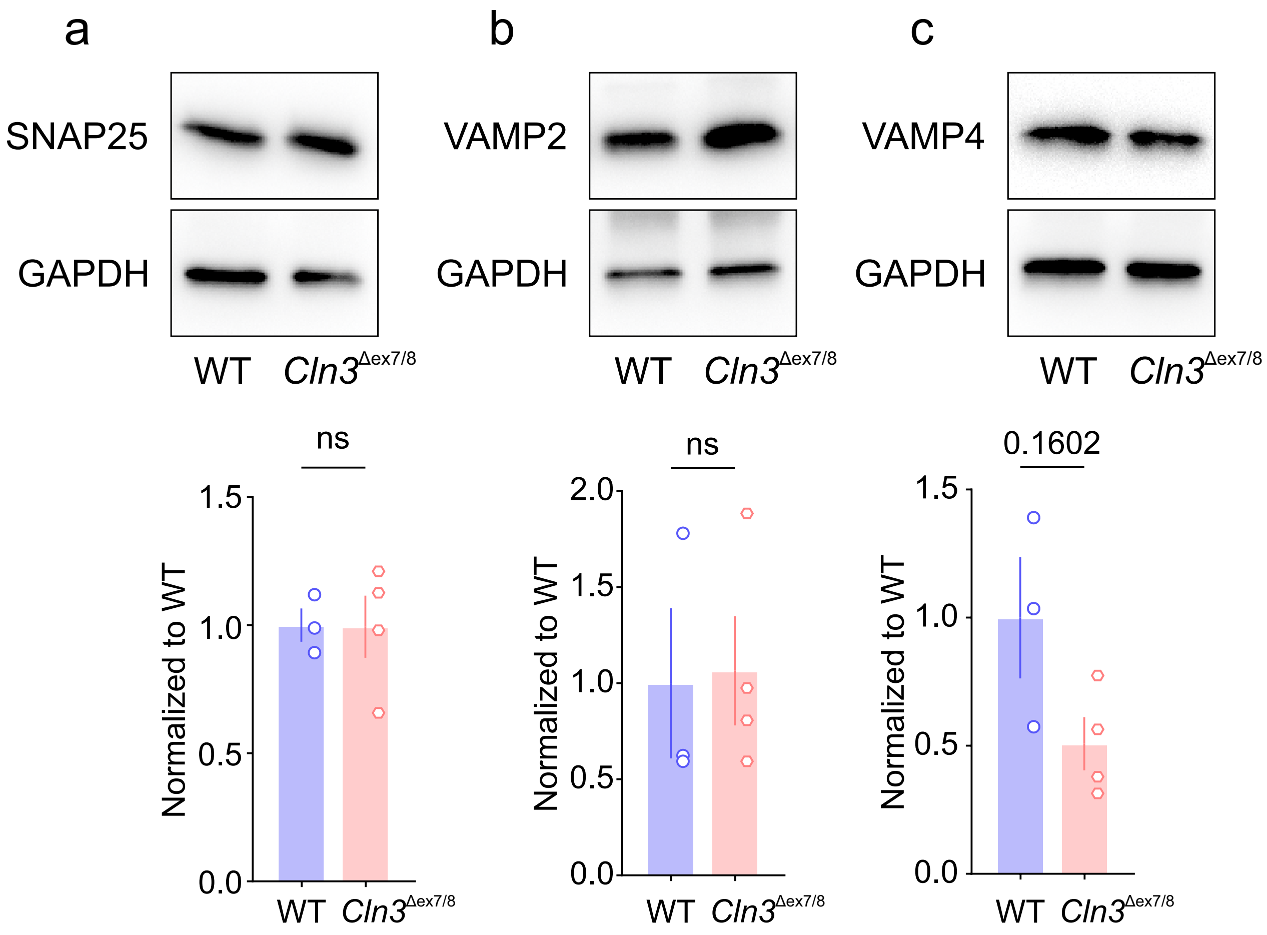

Fig. S5 Western blot analysis of presynaptic proteins in synaptosomal fractions from WT and 4-month-old *Cln3*$^{\Delta ex7/8}$ mice. Representative blots and quantification of (a) SNAP25, (b) VAMP2, and (c) VAMP4, normalized to GAPDH and expressed relative to WT (N=3,4 mice [WT, *Cln3*$^{\Delta ex7/8}$]). Data are presented as mean ± SEM; ns, not significant; p-value from Unpaired t-test.

Supplementary Figure S6

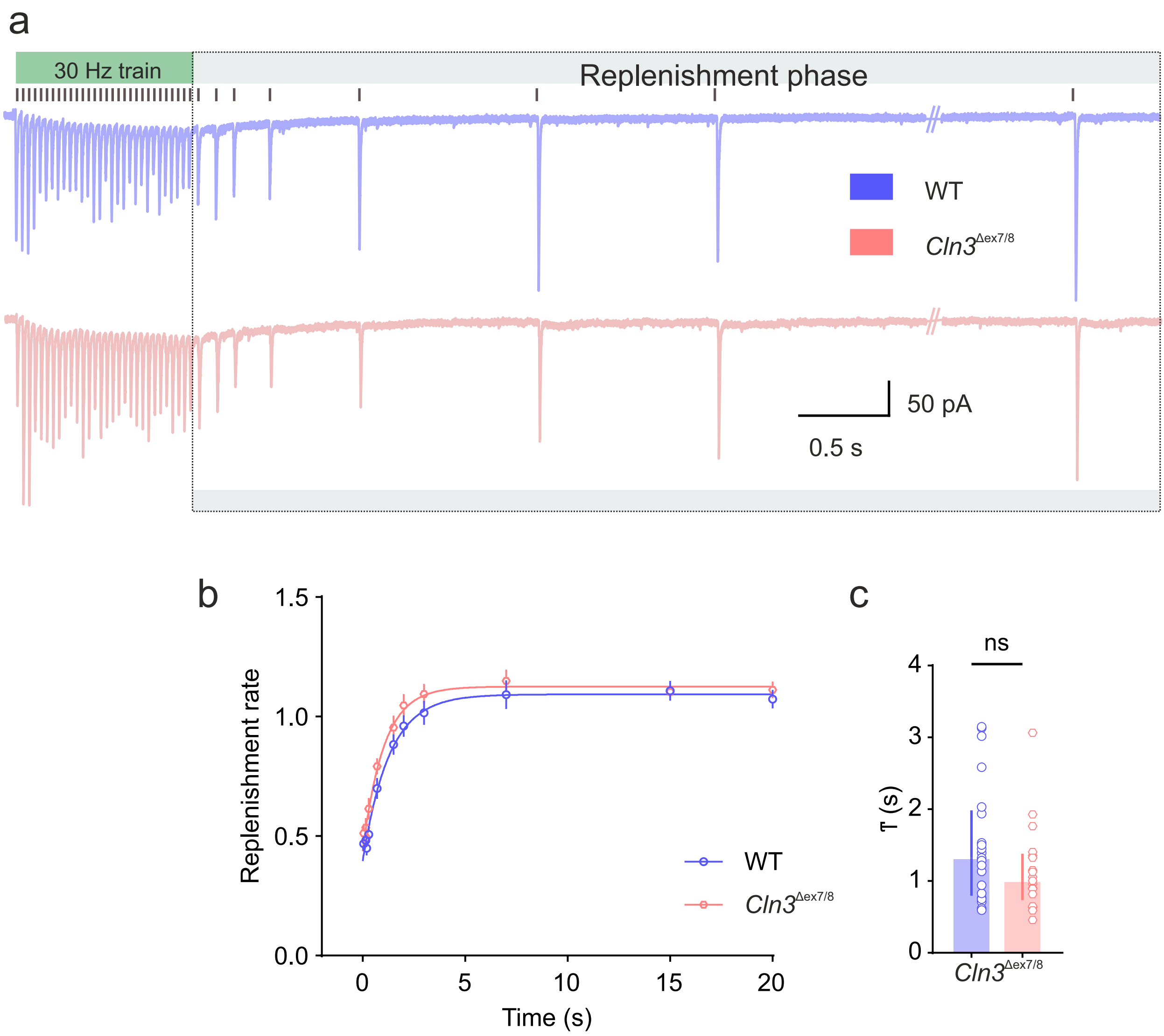

Fig. S6 RRP replenishment rate after pool depletion is unaffected at 4 months. (a) Representative trace of evoked EPSCs in response to 30 Hz perforant pathway stimulation, followed by single eEPSCs evoked at increasing inter-stimulus intervals (replenishment phase; dotted box, black lines above traces mark stimulus times). (b) Solid line represents single exponential decay fit of the data. (c) Summary bar plot of decay time constant (τ); n= 21, 21 cells; N= 4, 3 mice [WT, *Cln3*$^{\Delta ex7/8}$]. Data are median [IQR]; ns, not significant; linear mixed-effects model

Supplementary Figure S7

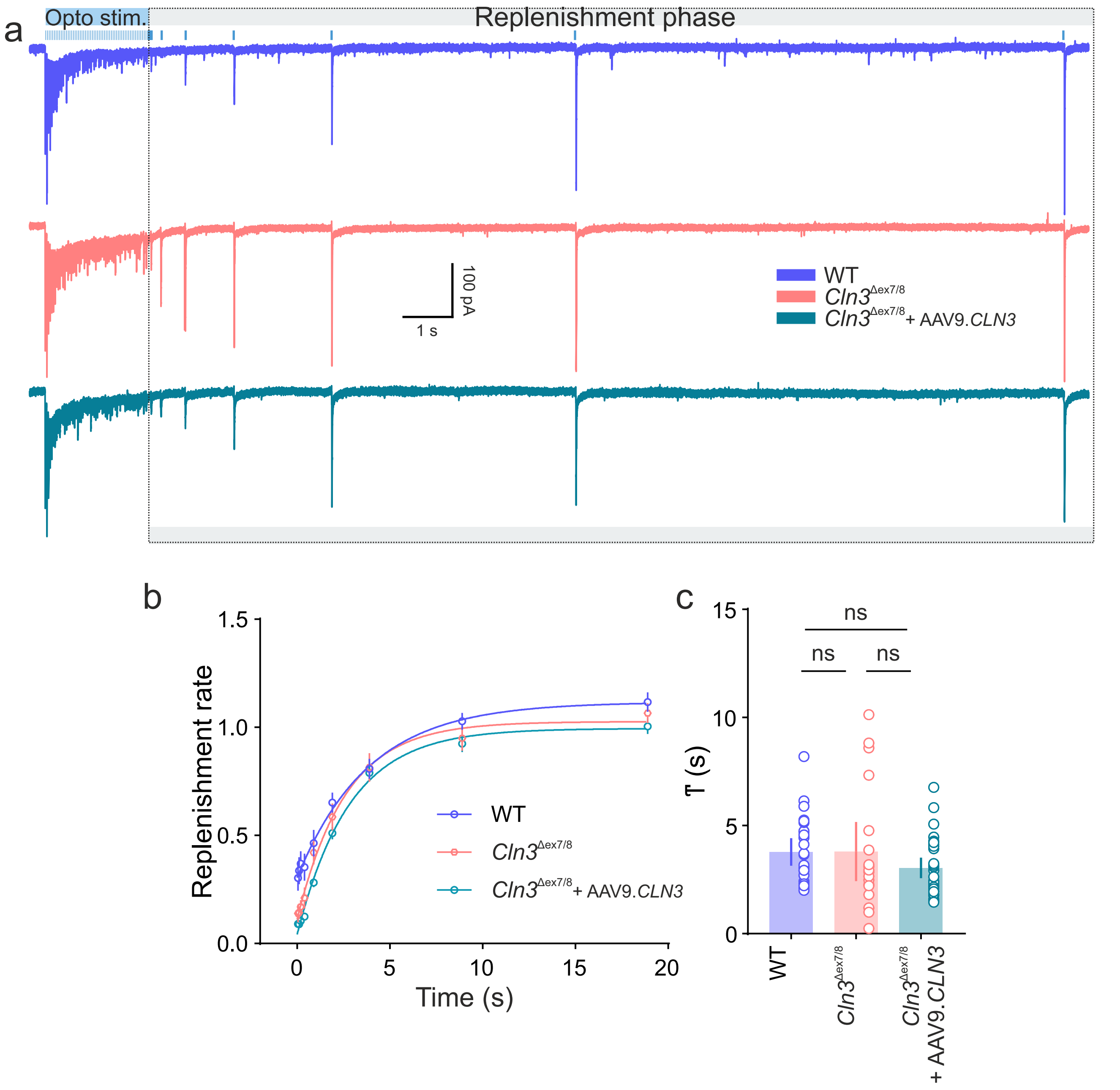

Fig. S7 Presynaptic re-expression of *CLN3* in 4-month-old *Cln3*$^{\Delta ex7/8}$ mice does not affect RRP replenishment rate. (a) Representative trace of evoked EPSCs in response to 30 Hz optical stimulation of the perforant pathway, followed by single eEPSCs evoked at increasing inter-stimulus intervals (replenishment phase; dotted box, blue lines above the traces indicate stimulus times). (b) Solid line represents single exponential decay fit of the data. (c) Summary bar plot of decay time constant (τ); n= 22, 20, 26 cells; N= 3, 3, 4 mice [WT, *Cln3*$^{\Delta ex7/8}$, *Cln3*$^{\Delta ex7/8}$ + AAV9.*CLN3*]. Data are median [IQR]; ns, not significant; linear mixed-effects model

Supplementary Figure S8

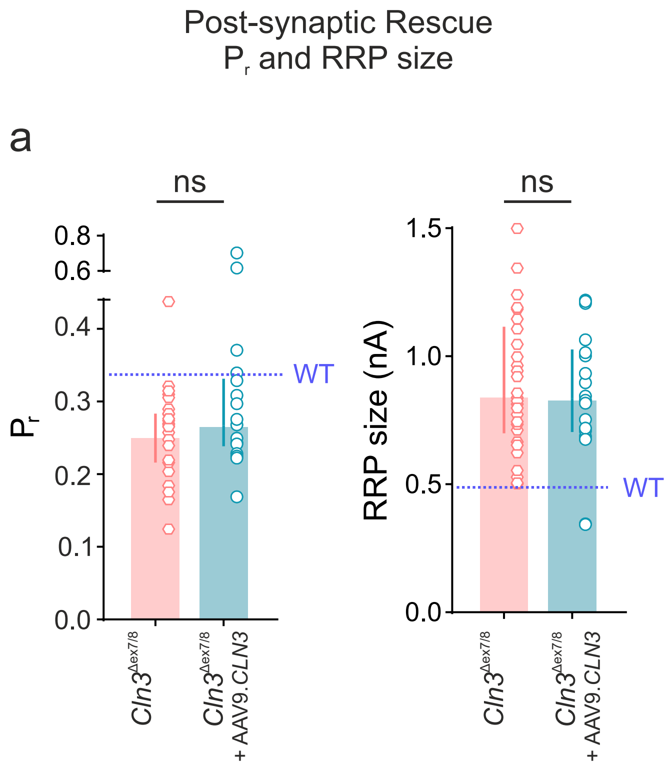

Fig. S8 Postsynaptic re-expression of *CLN3* does not improve presynaptic function in 4-month-old *Cln3*$^{\Delta ex7/8}$ mice. (a) Summary bar plots of release probability (P$_r$), readily releasable pool (RRP) size (n= 28, 19 cells; N= 4, 3 mice [*Cln3*$^{\Delta ex7/8}$, *Cln3*$^{\Delta ex7/8}$+ AAV9.*CLN3*]). Data are median [IQR] for P$_r$ and mean [95% CI] for RRP size; ns, not significant; linear mixed-effects model. Horizontal dashed line in blue above the data bars indicate WT values

Table 1. Intrinsic membrane properties of DG granule cells from WT and *Cln3*$^{ex7/8}$ mice at 3 weeks and 4 months of age

	Parameter	WT	*Cln3*$^{\Delta ex7/8}$	n (cells) & N (mice) (WT, *Cln3*$^{\Delta ex7/8}$)	Normality (Shapiro Wilk test)	Linear Mixed-effects model (genotype effect)
4 months	Input resistance (MΩ) median [P25 - P75]	179.7 [149.3 to 242.7]	196.6 [157.6 to 262]	n = 36, 43<br>N = 5, 5	No	Coefficient (β): -25.85<br>z-value= -1.37<br>p=0.170
	Resting membrane potential (mV) mean [95% CI]	-77.89 [-79.51 to -76.26]	-77.59 [-78.84 to -76.34]		Yes	Coefficient (β): -1.10<br>z-value= -0.557<br>p=0.577
3 weeks	Input resistance (MΩ) mean [95% CI]	425.90 [374.40 to 477.40]	384.80 [341.80 to 427.70]	n = 38, 33<br>N = 5, 4	Yes	Coefficient (β): 29.69<br>z-value= 0.453<br>p=0.649
	Resting membrane potential (mV) mean [95% CI]	-70.03 [-70.47 to -69.59]	-70.66 [-71.03 to -70.29]		Yes	Coefficient (β): 0.614<br>z-value= 1.454<br>p=0.145

	Parameter	Statistics			n (cells) & N (mice) (WT, *Cln3*$^{\Delta ex7/8}$)
4 months	Action Potential number vs current injection (pA)	Multiple Mann Whitney test (Multiple comparisons FDR: Two-stage set-up (Benjamini, Krieger and Yakutieli))			n = 26, 30<br>N = 4, 5
		Current step	160 pA	p=0.0100, q = 0.043	
			170 pA	p=0.0057, q = 0.043	
			180 pA	p=0.0117, q = 0.043	
3 weeks	Action Potential number vs current injection (pA)	Multiple Mann Whitney test (Multiple comparisons FDR: Two-stage set-up (Benjamini, Krieger and Yakutieli))			n = 27, 24<br>N = 5, 4
		Current step	50 pA	p=0.0136, q = 0.0642	
			60 pA	p=0.0063, q = 0.0632	
			70 pA	p=0.0169, q = 0.0642	

Action potential (AP) Kinetics (4-month-old)					
Parameter		WT	*Cln3*$^{\Delta ex7/8}$	Normality (Shapiro Wilk test)	Statistics (FDR: Two-stage set-up (Benjamini, Krieger and Yakutieli) n = 26, 30 cells; N = 4, 5 mice
4 months	AP threshold voltage (mV) median [P25 - P75]	-37.54 [-40.57 to -30.15]	-33.68 [-37.97 to -29.16]	No	Mann Whitney test p=0.126, q= 0.2646
	AP Amplitude (mV) median [P25 - P75]	91.50 [82.21 to 95.78]	88.13 [84.81 to 92.02]	No	Mann Whitney test p=0.263, q=0.3692
	AP Rise time (ms) median [P25 - P75]	0.37 [0.37 to 0.51]	0.50 [0.40 to 0.53]	No	Mann Whitney test p=0.0221, q=0.0696
	AP Decay time (ms) mean [95% CI]	1.81 [1.72 to 1.91]	1.84 [1.77 to 1.91]	Yes	Unpaired t test p=0.656, q=0.6888
	fast Afterhyperpolarization (fAHP) (mV) median [P25 - P75]	-17.38 [-19.87 to -15.29]	-15.59 [-19.82 to -12.57]	No	Mann Whitney test p=0.293, q=0.3692
	AP Half-width (ms) median [P25 - P75]	1.35 [1.23 to 1.41]	1.50 [1.24 to 1.73]	No	Mann Whitney test p=0.0151, q=0.0696

Action potential (AP) Kinetics (3-week-old)					
Parameter		WT	*Cln3*$^{\Delta ex7/8}$	Normality (Shapiro Wilk test)	Statistics (FDR: Two-stage set-up (Benjamini, Krieger and Yakutieli) n = 27, 24 cells; N = 5, 4 mice
3 weeks	AP threshold voltage (mV) median [P25 - P75]	-34.40 [-36.44 to -32.35]	-31.51 [-34.16 to -28.86]	Yes	Unpaired t test p=0.0841, q=0.2649
	AP Amplitude (mV) median [P25 - P75]	89.12 [85.31 to 91.79]	86.52 [77.86 to 88.90]	No	Mann Whitney test p=0.0289, q=0.1821
	AP Rise time (ms) median [P25 - P75]	0.40 [0.37 to 0.43]	0.37 [0.33 to 0.40]	No	Mann Whitney test p=0.389, q=0.4085
	AP Decay time (ms) mean [95% CI]	1.83 [1.70 to 1.97]	1.87 [1.77 to 2.00]	No	Mann Whitney test p=0.285, q=0.4085
	fast Afterhyperpolarization (fAHP) (mV) median [P25 - P75]	-18.61 [-20.61 to -16.60]	-19.82 [-21.32 to -18.32]	Yes	Unpaired t test p=0.329, q=0.4085
	AP Half-width (ms) median [P25 - P75]	1.49 [1.42 to 1.56]	1.43 [1.38 to 1.47]	Yes	Unpaired t test p=0.157, q=0.3297

Table 2. Synaptic transmission: mEPSC and mIPSC values of DG granule cells from WT and *Cln3*$^{ex7/8}$ mice at 3 weeks and 4 months of age

Parameter			WT	*Cln3*$^{\Delta ex7/8}$	n (cells) & N (mice) (WT, *Cln3*$^{\Delta ex7/8}$)	Normality (Shapiro Wilk test)	Linear Mixed-effects model (genotype effect)
mEPSC	4 months	Frequency (Hz) median [P25 - P75]	1.96 [1.61 to 2.79]	0.75 [0.53 to 1.28]	n = 17, 26; N = 3, 3	No	Coefficient (β): 1.426 z-value= 3.68 p=0.0002
		Amplitude (pA) mean [95% CI]	13.61 [12.45 to 14.76]	11.41 [10.48 to 12.34]		Yes	Coefficient (β): 2.22 z-value= 2.20 p=0.02
	3 weeks	Frequency (Hz) median [P25 - P75]	3.16 [2.27 to 4.61]	2.11 [1.55 to 2.66]	n = 28, 31; N = 3, 3	No	Coefficient (β): 1.248 z-value= 2.2 p=0.027
		Amplitude (pA) median [P25 - P75]	15.82 [13.57 to 17.36]	15.56 [13.90 to 16.84]		No	Coefficient (β): 0.23 z-value= 0.188 p=0.850

Parameter			WT	*Cln3*$^{\Delta ex7/8}$	n (cells) & N (mice) (WT, *Cln3*$^{\Delta ex7/8}$)	Normality (Shapiro Wilk test)	Linear Mixed-effects model (genotype effect)
mIPSC	4 months	Frequency (Hz) median [P25 - P75]	2.61 [1.82 to 3.43]	2.04 [1.48 to 2.74]	n = 31, 40; N = 5, 5	No	Coefficient (β): 0.31 z-value= 0.59 p=0.552
		Amplitude (pA) median [P25 - P75]	32.01 [24.90 to 37.59]	27.96 [21.48 to 33.58]		No	Coefficient (β): 2.23 z-value= 0.47 p=0.638
	3 weeks	Frequency (Hz) median [P25 - P75]	3.31 [2.07 to 4.56]	2.25 [1.68 to 4.67]	n = 26, 30; N = 4, 5	Yes	Coefficient (β): 0.70 z-value= 0.743 p=0.456
		Amplitude (pA) median [P25 - P75]	28.76 [20.53 to 38.94]	26.00 [20.89 to 33.82]		No	Coefficient (β): 3.28 z-value= 0.608 p=0.542

Table 3. AMPA/NMDA ratio values of DG granule cells from 4-month-old WT and *Cln3*$^{ex7/8}$ mice

Parameter	WT	*Cln3*$^{\Delta ex7/8}$	n (cells) & N (mice) (WT, *Cln3*$^{\Delta ex7/8}$)	Normality (Shapiro Wilk test)	Linear Mixed-effects model (genotype effect)
AMPA-NMDA ratio mean [95% CI]	1.86 [1.65 to 2.08]	1.18 [1.01 to 1.35]	n = 33, 42; N = 5, 6	Yes	Coefficient (β): 0.658 z-value= 3.31 p=0.0009
NMDAR decay τ (ms) mean [95% CI]]	36.45 [31.27 to 41.63]	40.86 [36.05 to 45.67]	n = 35, 40; N = 4, 4	Yes	Coefficient (β): 5.33 z-value= 0.944 p=0.344

Table 4. Dendritic spine density of DG granule cells from 4-month-old WT and *Cln3*$^{ex7/8}$ mice

Parameter	WT	*Cln3*$^{\Delta ex7/8}$	n (cells) & N (mice) (WT, *Cln3*$^{\Delta ex7/8}$)	Normality (Shapiro Wilk test)	Linear Mixed-effects model (genotype effect)
Spine count (per 10 µm) mean [95% CI]	18.8 [17.9 to 19.7]	16.05 [14.6 to 17.4]	n = 43, 39; N = 8, 5	Yes	Coefficient (β): 2.78 z-value= 3.4 p=0.0006
Spine count (per 10 µm) subtype - Mushroom mean [95% CI]	1.83 [1.38 to 2.67]	1.28 [0.74 to 2.12]		No	Coefficient (β): 0.882 z-value= 2.16 p=0.03
Spine count (per 10 µm) subtype - Thin mean [95% CI]	11.61 [10.09 to 13.49]	9.70 [7.34 to 13.2]		No	Coefficient (β): 2.10 z-value= 2.25 p=0.024
Spine count (per 10 µm) subtype - Stubby mean [95% CI]	4.53 [3.59 to 5.53]	4.31 [3.55 to 5.21]		No	Coefficient (β): 0.55 z-value= 1.52 p=0.128
Total dendritic length (mm) median [P25 - P75]	43.38 [37.50 to 49.26]	44.61 [38.99 to 50.22]	n = 17, 26; N = 3, 5	No	Coefficient (β): 54 z-value= 0.38 p=0.701

Table 5. Readily-releasable pool (RRP) values of DG granule cells from 4-month-old WT and *Cln3*$^{ex7/8}$ mice

Parameter	WT	*Cln3*$^{\Delta ex7/8}$	n (cells) & N (mice) (WT, *Cln3*$^{\Delta ex7/8}$)	Normality (Shapiro Wilk test)	Linear Mixed-effects model (genotype effect)
RRP size (nA)<br>median [P25 - P75]	0.629<br>[0.498 to 0.782]	0.815<br>[0.62 to 1.02]	n = 31, 25;<br>N = 4, 3	No	Coefficient (β): 0.186<br>z-value= 3.07<br>p=0.002
P_r (release probability)<br>mean [95% CI]	0.34<br>[0.31 to 0.38]	0.27<br>[0.24 to 0.31]		Yes	Coefficient (β): 0.06<br>z-value= 2.87<br>p=0.004
Slope<br>median [P25 - P75]	0.10<br>[0.08 to 0.13]	0.10<br>[0.08 to 0.14]		No	Coefficient (β): 0.01<br>z-value= 0.940<br>p=0.346
Replenishment rate τ (ms)<br>median [P25 - P75]	1.32<br>[0.79 to 1.98]	1.007<br>[0.733 to 1.38]		No	Coefficient (β): 0.364<br>z-value= 1.48<br>p=0.138

Table 6. Hippocampal LTP in 4-month-old WT and *Cln3*$^{ex7/8}$ mice

	Parameter	WT	*Cln3*$^{\Delta ex7/8}$	n (cells) & N (mice) (WT, *Cln3*$^{\Delta ex7/8}$)	Linear Mixed-effects model (genotype effect)
Induced	First 10 min<br>EPSC peak %<br>mean [95% CI]	207.9<br>[162.1 to 253.8]	183.4<br>[138.0 to 228.8]	n = 13, 14;<br>N = 6, 5	Coefficient (β): 23.96<br>z-value= 0.72<br>p=0.467
	Last 10 min<br>EPSC peak %<br>mean [95% CI]	181.1<br>[140.3 to 221.9]	183.8<br>[129.6 to 238.0]		Coefficient (β): 3.20<br>z-value= 0.088<br>p=0.929
Control	First 10 min<br>EPSC peak %<br>mean [95% CI]	68.68<br>[57.50 to 79.86]	64.99<br>[52.14 to 77.83]		Coefficient (β): 3.69<br>z-value= 0.450<br>p=0.652
	Last 10 min<br>EPSC peak %<br>mean [95% CI]	72.13<br>[54.77 to 89.48]	67.08<br>[54.87 to 79.29]		Coefficient (β): 5.02<br>z-value= 0.532<br>p=0.594

Table 7. Presynaptic properties recorded from DG-GCs after AAV9-mediated *CLN3* re-expression in presynaptic perforant pathway projections from Entorhinal cortex to DG in 4-month-old *Cln3*$^{ex7/8}$ mice

	WT	*Cln3*$^{\Delta ex7/8}$	*Cln3*$^{\Delta ex7/8}$ + AAV9.*CLN3*	Linear Mixed-effects model (genotype effect)	β	z-value	p-value
n (cells)	22	20	26				
N (mice)	3	3	4				
RRP size (nA) median [P25 - P75]	0.477 [0.352 to 0.702]	0.920 [0.598 to 1.12]	0.632 [0.497 to 0.770]	WT vs *Cln3*$^{\Delta ex7/8}$	0.434	2.24	0.025
				WT vs *Cln3*$^{\Delta ex7/8}$ + AAV9.*CLN3*	0.096	1.10	0.268
				Cln3$^{\Delta ex7/8}$ vs *Cln3*$^{\Delta ex7/8}$ + AAV9.*CLN3*	0.327	2.52	0.011
P_r (release probability) median [P25 - P75]	0.277 [0.240 to 0.396]	0.186 [0.136 to 0.262]	0.257 [0.233 to 0.338]	WT vs *Cln3*$^{\Delta ex7/8}$	0.116	2.54	0.010
				WT vs *Cln3*$^{\Delta ex7/8}$ + AAV9.*CLN3*	0.039	0.974	0.329
				Cln3$^{\Delta ex7/8}$ vs *Cln3*$^{\Delta ex7/8}$ + AAV9.*CLN3*	0.062	2.42	0.015
Slope median [P25 - P75]	0.013 [0.009 to 0.018]	0.012 [0.009 to 0.017]	0.010 [0.007 to 0.014]	WT vs *Cln3*$^{\Delta ex7/8}$	0.0047	0.131	0.895
				WT vs *Cln3*$^{\Delta ex7/8}$ + AAV9.*CLN3*	0.0036	1.57	0.114
				Cln3$^{\Delta ex7/8}$ vs *Cln3*$^{\Delta ex7/8}$ + AAV9.*CLN3*	0.0027	0.982	0.325
Replenishment rate τ (ms) median [P25 - P75]	3.41 [2.38 to 4.84]	2.85 [2.21 to 4.76]	2.44 [1.97 to 4.17]	WT vs *Cln3*$^{\Delta ex7/8}$	0.334	0.552	0.580
				WT vs *Cln3*$^{\Delta ex7/8}$ + AAV9.*CLN3*	0.516	1.31	0.188
				Cln3$^{\Delta ex7/8}$ vs *Cln3*$^{\Delta ex7/8}$ + AAV9.*CLN3*	0.405	0.764	0.444

Table 8. Pre- and postsynaptic properties recorded from DG-GCs after AAV9-mediated *CLN3* re-expression in postsynaptic granule cells of DG in 4-month-old *Cln3*$^{ex7/8}$ mice

Parameter	*Cln3*$^{\Delta ex7/8}$	*Cln3*$^{\Delta ex7/8}$ + AAV9.*CLN3*	n (cells) & N (mice) (*Cln3*, *Cln3*$^{\Delta ex7/8}$ + AAV9.*CLN3*)	Normality (Shapiro Wilk test)	Linear Mixed-effects model (genotype effect)
AMPA-NMDA ratio<br>median [P25 - P75]	1.43<br>[0.98 to 1.76]	1.73<br>[1.39 to 2.33]	n = 33, 45; N = 5, 5	No	Coefficient (β): 0.595<br>z-value= 2.44<br>p=0.0145

	Parameter	*Cln3*$^{\Delta ex7/8}$	*Cln3*$^{\Delta ex7/8}$ + AAV9.*CLN3*	n (cells) & N (mice) (*Cln3*, *Cln3*$^{\Delta ex7/8}$ + AAV9.*CLN3*)	Normality (Shapiro Wilk test)	Linear Mixed-effects model (genotype effect)
Presynaptic metrics after postsynaptic rescue	mEPSC Frequency (Hz)<br>median [P25 - P75]	0.40<br>[0.179 to 0.738]	0.33<br>[0.267 to 0.506]	n = 25, 22; N = 4, 3	No	Coefficient (β): 0.063<br>z-value= 0.627<br>p=0.530
	mEPSC Amplitude (pA)<br>mean [95% CI]	12.42<br>[11.84 to 13.01]	13.82<br>[12.62 to 15.01]		Yes	Coefficient (β): 1.384<br>z-value= 2.24<br>p=0.0245
	RRP size (nA)<br>mean [95% CI]	0.893<br>[0.797 to 0.989]	0.849<br>[0.721 to 0.976]	n = 28, 19; N = 4, 3	Yes	Coefficient (β): 0.0329<br>z-value= 0.291<br>p=0.770
	P_r (release probability)<br>median [P25 - P75]	0.253<br>[0.216 to 0.283]	0.268<br>[0.238 to 0.316]		No	Coefficient (β): 0.014<br>z-value= 0.806<br>p=0.420
